# Burnout in gastroenterology: Better awareness and research are needed

**DOI:** 10.1177/2050640620964643

**Published:** 2020-10-06

**Authors:** John Ong, Carla Swift, Sharon Ong

**Affiliations:** 1 East of England Gastroenterology Training Programme, Cambridge, UK; 2 Department of Engineering, University of Cambridge, Cambridge, UK; 3 Department of Medicine, National University of Singapore, Singapore; 4 Department of Surgical Intensive Care, Division of Anaesthesiology and Perioperative Sciences, Singapore General Hospital, Singapore; 5 Department of Surgical Intensive Care, Division of Anaesthesiology and Perioperative Sciences, Sengkang General Hospital, Singapore

We read with interest the article by Buscarini et al.^1^ Although burnout is defined as an ‘occupational phenomenon’ rather than a mental health illness by the World Health Organization, we fully agree that it remains an important issue to address in gastroenterology, especially in the coronavirus disease 2019 era. Previous large-scale burnout studies in gastroenterology have originated mostly from the USA (pre-pandemic), and the current prevalence in the field remains poorly understood globally. Also, previous results cannot be reliably extrapolated across countries because of differences in health-care systems, working environment, study population, burnout detection tools and burnout criteria.^2^ Therefore, the scale of the problem must first be understood locally to compel organisational change.

In managing burnout, we believe greater awareness of the syndrome is also needed amongst gastroenterologists to enable self-reporting, since a gold standard detection tool does not exist currently. Symptoms of emotional exhaustion and depersonalisation are telling of burnout. However, abnormal symptoms in only one domain can be reflective of an individual’s personality type.^3^ Symptoms of low personal accomplishment alone correlates poorly with burnout. These may exist in unaffected individuals and often have poor factor loading in detection tools.^3^,^4^ Nonetheless, the most extensively validated and commonly used tool to identify burnout in medical professionals – the 22-item Maslach Burnout Inventory (MBI) – has its limitations and can be costly as well as time-consuming to administer. Therefore, authorities must be prepared to allocate the time and resources to assess individuals holistically in order to achieve meaningful change. Abbreviated versions of the MBI can be inaccurate.^5^ So, we and other researchers advise against its use.

If in doubt, or when burned-out individuals are identified, we also believe they should be referred to mental well-being support services where possible, since burnout may co-exist with undiagnosed mental health conditions such as depression. As such, comprehensive mental-health assessments should not be overlooked or replaced by ‘peer support’ alone. That said, support services must be well advertised and signposted for individuals to access. In a recent pilot study of gastroenterology trainees in the East of England, even though 35.3% had burnout or were at high risk of burnout, 51.6% of all trainees were unaware of available mental well-being services that existed within the training programme.^6^

In closing, we fully support Buscarini et al.’s call for better safeguards against burnout in gastroenterology. In addition, more research on burnout is also needed within the specialty.
